# Autoimmune Polyendocrine Syndrome 3 Onset with Severe Ketoacidosis in a 74-Year-Old Woman

**DOI:** 10.1155/2015/960615

**Published:** 2015-03-02

**Authors:** Stefano Benedini, Antonietta Tufano, Elena Passeri, Marco Mendola, Livio Luzi, Sabrina Corbetta

**Affiliations:** ^1^Department of Biomedical Sciences for Health, Università degli Studi di Milano, Via Morandi 30, San Donato Milanese, 20097 Milan, Italy; ^2^Metabolism Research Center, IRCCS Policlinico San Donato, Via Morandi 30, San Donato Milanese, 20097 Milan, Italy; ^3^Endocrinology Unit, IRCCS Policlinico San Donato, Via Morandi 30, San Donato Milanese, 20097 Milan, Italy; ^4^Endocrinology and Metabolic Diseases, IRCCS Policlinico San Donato, Via Morandi 30, San Donato Milanese, 20097 Milan, Italy

## Abstract

Type 1 diabetes mellitus (T1D), autoimmune thyroid disease, and autoimmune gastritis often occur together forming the so-called autoimmune polyendocrine syndrome type 3 (APS3). We here report a clinical case of a 74-year-old woman who presented for the first time with severe hyperglycemia and ketoacidosis diagnosed as T1D. Further clinical investigations revealed concomitant severe hypothyroidism with autoimmune thyroid disease and severe cobalamin deficiency due to chronic atrophic gastritis. The diagnosis of type 1 diabetes mellitus was confirmed by the detection of autoantibodies against glutamic acid decarboxylase 65, islet cell antibodies, and anti-insulin autoantibodies. Anti-thyroperoxidase, anti-thyroglobulin, and anti-gastric parietal cell antibodies were also clearly positive. The case emphasized that new onset diabetic ketoacidosis, hypothyroidism, and cobalamin deficiency may simultaneously occur, and one disease can mask the features of the other, thereby making diagnosis difficult. It is noteworthy that an APS3 acute episode occurred in an asymptomatic elder woman for any autoimmune diseases.

## 1. Introduction

Type 1 diabetes mellitus (T1D), autoimmune thyroid disease (ATD), and autoimmune atrophic gastritis (AAG) often occur together forming the so-called autoimmune polyendocrine syndrome (APS) type 3. Thyroid autoimmunity is evident in up to one-third and gastric autoimmunity in up to a quarter of patients with T1D [1]. T1D has historically been considered a predominant disorder of children and young adults: the disease has been commonly referred to as juvenile diabetes because it shows a peak of occurrence at 10–14 years. However, recent studies support a different model in which the disease may occur at any age. Such patients are best identified by the presence of anti-islet autoantibodies, in particular autoantibodies against glutamic acid decarboxylase 65 (GAD65) [2].

Nonetheless the most common presentation of APS consists in an autoimmune thyroid disease without adrenal insufficiency and another associated autoimmune disease such as T1D, pernicious anemia, vitiligo, or alopecia that usually develops in middle-aged women.

Here a case of APS3 presenting with ketoacidosis in an otherwise healthy elder woman was described.

## 2. Case Presentation

The female patient, aged 74 years, weighed 45 kg with a BMI of 20.5 Kg/m^2^. She was admitted through the Emergency Department at the IRCCS Policlinico San Donato (San Donato Milanese, Italy) for severe drowsiness and unresponsiveness. Symptoms promptly occurred after a flu-like syndrome. Her vital signs were significant for mild hypotension. Her physical exam revealed severe dehydration, though the patient was relatively well nourished. Her admission laboratory values were significant for blood glucose level of 61.4 mmol/L and pH of 7.2 with bicarbonate of 19 mmol/L. Urine analysis showed massive glycosuria and ketonuria (Table 1). Brain computed tomography showed no noticeable space occupying cerebral lesion or recent density abnormalities of the white or gray matter. Rehydration and insulin intravenous infusion restored patient's consciousness within 3-4 hours without neuropsychiatric sequelae.

She was admitted to the Endocrine Unit of the IRCCS Policlinico San Donato where metabolic variables were stabilized within 3 days. Endocrine screening unmasked concomitant severe hypothyroidism (serum TSH 102 *μ*U/mL, free T4 0.15 ng/mL). The clinical signs associated with hypothyroidism were elevated total cholesterol (248 mg/dL) and creatine kinase (186 UI/L, n.v <176). Ultrasound imaging of the neck showed a thyroid gland of reduced volume and with hypoechoic features of thyroiditis. L-thyroxine replacement was started following the check of conserved adrenal secretion (ACTH pg/mL, cortisol *μ*g/dL). Further biochemical evaluation revealed mild anemia (haemoglobin 11 g/dL; n.v. 12–16) with macrocytosis (mean red cell volume 100.5 fL) associated with reduced serum folates (5 ng/mL; n.v. 5–19) and vitamin B12 (199 pg/mL; n.v. 191–663). Endoscopy diagnosed severe atrophic gastritis that was histologically confirmed by multiple biopsies. Cobalamin and folates were replaced. The patient significantly improved over a 14-day long hospitalization. She was discharged in good general condition.

An autoimmunity screening was performed: autoantibodies against glutamic acid decarboxylase (GAD) have positive results as well as anti-islet cell autoantibodies (ICA) and anti-insulin autoantibodies (IAA) (Table 2). Moreover, anti-peroxidase (AbTPO) and anti-thyroglobulin (AbTg) autoantibodies (Table 2) as well as anti-parietal cells autoantibodies (APCA) were detected in the patient's serum.

The case here reported emphasizes that new onset diabetic ketoacidosis, hypothyroidism, and cobalamin deficiency may occur, and one disease can mask the features of the other, thereby making diagnosis a clinical challenge. It is noteworthy that an utterly asymptomatic patient to any autoimmune diseases had a APS3 acute episode (i.e., hyperglycemia, severe hypothyroidism, and cobalamin deficiency) in her elder age. Overt diabetes onset with typical signs of polyuria and polydipsia was likely delayed by the concomitant low metabolic basal rate secondary to the severe unrecognized hypothyroidism and malabsorption. In this context, the flu-like episode was sufficient to precipitate the impaired metabolic homeostasis. Indeed, acute infections are known to usually drop myxedematous coma.

Moreover, the present case reminds us that autoimmunity might arise in the elderly. In a previous study on T1D Northern Italian patients, the prevalence of diabetes-related autoantibodies was higher in the 21–40-year-old group compared to the 41–72-year-old group and these antibodies have higher results in females than in males [3]. GAD, ICA, and IAA were clearly detectable in the serum of the reported patient. Autoimmune thyroid disorders are the most prevalent immunological diseases in T1D patients [4]. The prevalence of positive AbTPO has been reported in about 80% of T1D patients with elevated TSH levels and in 10–20% in euthyroid T1D individuals. Most patients have subclinical disease, and the development of diabetes usually precedes the diagnosis of hypothyroidism [5]. Due to the increased prevalence of thyroid dysfunction in T1D subjects, regular screening of TSH is recommended [4]. Atrophic gastritis is present in about 25–40% of patients with T1D, whereas in thyroiditis the prevalence of atrophic gastritis is present in approximately one-third of cases. Therefore, the occurrence of multiorgan autoimmune involvement is not unusual though concomitant related insufficiency, as occurred in the present case, is surprising and, more importantly, might be life-threatening. The present case report supports the inclusion of serum TSH and cobalamin determinations among the hormone tests at admission of diabetic ketoacidotic elder patients.

## Figures and Tables

**Table 1:** Anthropometrical and clinical characteristics of the patient upon admission to hospital and at discharge.

Variable	Emergency Department	At hospital discharge
Weight (kg)	45	47
Height (cm)	148	148
BMI (kg/m^2^)	20.5	21.4
Blood glucose (mmol/L)	61.39	6.83
Insulin (*μ*U/mL)	1.1	0.7
C-peptide (ng/mL)	1.1	0.8
Urinary glucose (mg/dL)	1000	0
Blood K^+^ (mEq/L)	4.67	3.93
Blood Na^+^ (cm)	141	137
TSH (*μ*UI/mL)	102	10.2
FT3 (pg/mL)	0.6	2.3
FT4 (ng/dL)	0.15	1.01

**Table 2:** Organ specific autoantibodies evaluated during hospitalization.

Organ specific autoantibodies	
GADA (glutamic acid decarboxylase antibodies)	Positive
ICA (islet cell antibodies)	Positive
IAA (insulin autoantibodies)	Positive
IA2 (tyrosine-phosphatase autoantibodies)	Negative
Znt8 (autoantibodies to the islet-specific zinc transporter isoform 8)	Negative
TPO (antibodies anti-peroxidase)	Positive
Ab TG (antibodies anti-thyroglobulin)	Positive
PCA (antibodies anti-parietal cells)	Positive
ACA (adrenal cortex antibodies)	Negative
AGA (anti-gliadin antibodies)	Negative
EMA (anti-endomysium antibody)	Negative
